# Psychometric properties of the Vulnerability to Abuse Screening Scale for screening abuse of older adults

**DOI:** 10.1590/S1518-8787.2017051006839

**Published:** 2017-03-29

**Authors:** Raquel Batista Dantas, Graziella Lage Oliveira, Andréa Maria Silveira

**Affiliations:** I Programa de Mestrado Profissional em Promoção da Saúde e Prevenção da Violência. Hospital da Polícia Militar de Minas Gerais. Faculdade de Medicina. Universidade Federal de Minas Gerais. Belo Horizonte, MG, Brasil; II Departamento de Medicina Preventiva e Social. Faculdade de Medicina. Universidade Federal de Minas Gerais. Belo Horizonte, MG, Brasil

**Keywords:** Elder Abuse, Domestic Violence, Psychometrics, Reproducibility of Results, Validation Studies

## Abstract

**OBJECTIVE:**

Adapt and evaluate the psychometric properties of the Vulnerability to Abuse Screening Scale to identify risk of domestic violence against older adults in Brazil.

**METHODS:**

The instrument was adapted and validated in a sample of 151 older adults from a geriatric reference center in the municipality of Belo Horizonte, State of Minas Gerais, in 2014. We collected sociodemographic, clinical, and abuse-related information, and verified reliability by reproducibility in a sample of 55 older people, who underwent re-testing of the instrument seven days after the first application. Descriptive and comparative analyses were performed for all variables, with a significance level of 5%. The construct validity was analyzed by the principal components method with a tetrachoric correlation matrix, the reliability of the scale by the weighted Kappa (Kp) statistic, and the internal consistency by the Kuder-Richardson estimator formula 20 (KR-20).

**RESULTS:**

The average age of the participants was 72.1 years (DP = 6.96; 95%CI 70.94–73.17), with a maximum of 92 years, and they were predominantly female (76.2%; 95%CI 69.82–83.03). When analyzing the relationship between the scores of the Vulnerability to Abuse Screening Scale*,* categorized by presence (score > 3) or absence (score < 3) of vulnerability to abuse, with clinical and health conditions, we found statistically significant differences for self-perception of health (p = 0.002), depressive symptoms (p = 0.000), and presence of rheumatism (p = 0.003). There were no statistically significant differences between sexes. The Vulnerability to Abuse Screening Scale acceptably evaluated validity in the transcultural adaptation process, demonstrating dimensionality coherent with the original proposal (four factors). In the internal consistency analysis, the instrument presented good results (KR-20 = 0.69) and the reliability via reproducibility was considered excellent for the global scale (Kp = 0.92).

**CONCLUSIONS:**

The Vulnerability to Abuse Screening Scale proved to be a valid instrument with good psychometric capacity for screening domestic abuse against older adults in Brazil.

## RESUMO

**OBJETIVO:**

Adaptar e avaliar as propriedades psicométricas da Escala Triagem de Vulnerabilidade ao Abuso (*Vulnerability to Abuse Screening Scale –* VASS) para identificar risco de violência doméstica contra idosos no Brasil.

**MÉTODOS:**

O instrumento foi adaptado e validado em uma amostra de 151 idosos de um centro de referência do idoso, do município de Belo Horizonte, MG, em 2014. Foram coletadas informações sociodemográficas, clínicas e relacionadas a abusos. A confiabilidade via reprodutibilidade foi verificada em uma amostra de 55 idosos, submetidos ao reteste do instrumento sete dias após a primeira aplicação. Foram realizadas análises descritivas e comparativas para todas as variáveis, com nível de significância de 5%. A validade de construto foi analisada pelo método de componentes principais com matriz de correlação tetracórica, a confiabilidade da escala pela estatística Kappa ponderado (Kp) e a consistência interna pelo estimador Kuder-Richardson fórmula 20 (KR-20).

**RESULTADOS:**

A idade média dos participantes foi 72,1 anos (DP = 6,96; IC95% 70,94–73,17), com máxima de 92 anos, e o sexo foi predominantemente feminino (76,2%; IC95% 69,82–83,03). Ao analisar a relação entre os escores da Escala Triagem de Vulnerabilidade ao Abuso, categorizados em presença (índice > 3) ou ausência (índice < 3) de vulnerabilidade para abuso, com condições clínicas e de saúde, encontramos diferenças estatisticamente significantes para autopercepção de saúde (p = 0,002), sintomas depressivos (p = 0,000) e presença de reumatismo (p = 0,003). Em relação ao sexo, não houve diferença estatisticamente significante. A Escala Triagem de Vulnerabilidade ao Abuso comportou-se de forma aceitável na avaliação da validade no processo de adaptação transcultural, demonstrando dimensionalidade coerente com a proposta original (quatro fatores). Na análise da consistência interna, o instrumento apresentou bons resultados (KR-20 = 0,69) e a confiabilidade via reprodutibilidade foi considerara excelente para a escala global (Kp = 0,92).

**CONCLUSÕES:**

A Escala Triagem de Vulnerabilidade ao Abuso mostra-se como instrumento válido e com boas capacidades psicométricas para o rastreio de abuso doméstico contra idosos no contexto brasileiro.

## INTRODUCTION

Violence is a worldwide phenomenon and a relevant public order issue. For older adults, the literature presents consolidated evidence of mortality by violence associated with falls and transportation accidents^1^ ^-^ ^3^. However, we know little of the reality of violence such as abuse that occurs mainly in the home environment^9^ ^,^ ^12^ ^,^ ^14^ ^,^ ^a^ .

This situation demands the construction and validation of tools for screening violence against elderly people^4^ ^,^ ^16^, especially in transcultural studies, allowing the results from different samples to be compared^17^.

There are currently two instruments for screening domestic violence against older adults, validated for Brazilian culture and including psychometric studies^16^: the Hwalek-Sengstock Elder Abuse Screening Test (H-S/EAST)^19^ and the Caregiver Abuse Screen (CASE)^20^, which collects information on potential abuse by caregivers.

In addition, the Vulnerability to Abuse Screening Scale (VASS)^16^ ^,^ ^23^ was considered eligible for validation in Brazil because it is simple and self-administered. This scale consists of 12 dichotomous items. Its cut-off point is interpreted as high vulnerability to violence, at a score of three or higher. Despite the VASS having questions from the H-S/EAST, in its psychometric analysis, these items behaved differently when allocated to different factors than those presented in the H-S/EAST study. Thus, the VASS consists of a new construct, and is therefore an alternative instrument to its predecessor.

The decision to analyze VASS was also based on the premise that the scale has a variable validated for Brazilian culture in Conflict Tactics Scales (CTS)^6^ ^,^ ^11^ that could suggest the occurrence of violence by an intimate partner, and an item related to the feeling of fear of someone in the family, which could explain the general construct of vulnerability to abuse, especially from people close to the older adults.

In this light, this study aimed to transculturally adapt the VASS – Vulnerability to Abuse Screening Scale for older adults to Portuguese and analyze its psychometric properties of validity and reliability.

## METHODS

We performed a cross-sectional study grounded in the procedures for transcultural adaptation proposed by Reinchenhem and Moraes^18^, Herdmam et al.^7^, and Beaton et al.^2^, as well as equivalence analyses of concepts and items; semantics; operations; and measurements.

A team of specialists evaluated the equivalence of concepts and items by assessing the pertinence of the terms and concepts used in the original instrument and the impact of their meanings in Brazilian culture. They also explored the adaptation of items from the original instrument in relation to their capacity of representing these dimensions in the population^14^ ^,^ ^18^.

The semantic equivalence process involved a grammar and vocabulary analysis, where we verified if the translation expresses the same concept as the original instrument and is adapted to the local reality^2^ ^,^ ^7^ ^,^ ^18^. We did so in five steps.

In the first, bilingual translators performed two translations (T1 and T2), which moved on to the second step for reverse translation (RT1 and RT2), sent to two medical translators, blindly and independently, one of whom was a sworn translator and the other from the same country as the original instrument. In the third step, we forwarded the reverse translations to a fifth bilingual evaluator, who verified the general and referential meanings of the terms and expressions in the scale.

The connotative (general) meaning was classified into four categories: unchanged, slightly changed, drastically changed, and completely changed. The referential meaning was analyzed according to the Visual Analogue Scale*,* for which equivalence is judged from zero to 100%. Thus, the greater the relationship of literal correspondence between the translated terms and the original version, the higher the equivalence to the referential meaning. In the fourth step, five specialized professionals, including the researchers and one medical geriatrician, formulated a final consolidated version in Portuguese, incorporating the necessary changes.

Lastly, the synthesized version was applied via a focal group to a sample of older adults to verify the acceptability and comprehension of the instrument. We read each item to the participants, who expressed understanding and suggested changes. The reformulation of items was based on a percentage equal to or higher than 15% of interviewees who did not understand the questions.

The dimensional structure of the VASS was measured by exploratory factorial analysis with the extraction of principal components by the tetrachoric correlation matrix^10^. The model and the factorial charges were adjusted by *varimax* orthogonal rotation^5^.

The internal consistency was estimated by the Kuder-Richardson coefficient, formula 20 (KR-20), and by intra-observer reliability through concordance by simple Kappa (K) and Kappa with quadratic weighting (Kp), where the latter were interpreted as recommended by Shrout^25^. The KR-20 coefficients were obtained on three levels: for the general scale, for the construct (factor) dimension, and after the sequential subtraction of each item from the scale (KR-20_KR-1_). We effected intra-observer reliability in a random simple sample of 55 older adults, to whom we reapplied the instrument approximately seven days after the first interview. Simple Kappa values were estimated for each item and the weighted Kappa for the general score, given the sum of the points in the 12 items of the scale, and for the factors extracted in the factorial analysis.

We analyzed the degree to which the data was adjusted for the factorial analysis via Kaiser-Meyer-Olkin (KMO)^8^ and Barthlet Sphericity^5^, considering a significance level of 0.05.

We collected data from February to May 2014 at the Reference Center for Elderly People of Belo Horizonte (Minas Gerais). In addition to the VASS, we used a questionnaire consisting of variables of the following types: sociodemographic; clinical (self-perception of health, chronic-degenerative diseases, immune state, polypharmacy, and use of health services); functionality (Lawton and Brody Scale); mental health and mood (Mini Mental State Exam [MEEM]^3^ and the Geriatric Depression Scale [GDS] 15 items^1^); variables of physical, psychological, sexual, and financial abuse; use of alcoholic beverages by family member; visit of family or friends in the last 30 days; difficulties managing money; and financial loans taken out in the last year. The inclusion criteria were: adults aged 60 years or older, with sufficient speech, hearing, and cognitive abilities (considering their MEEM^20^ performance). There were no exclusions.

The sample size was defined by the criteria suggested by Hair^5^, with five to ten individuals for each item of the instrument, reaching a total of 151 older adults^5^. We used the Statistical Package for the Social Sciences (version 20) and the R program, version 3.03. The significance level considered for all tests was 0.05.

This study was approved by the Research Ethics Committee of the Federal University of Minas Gerais (Certificate of Presentation for Ethical Appreciation [CAAE] 02235212.2.0000.5149). All participants signed the free and informed consent term.

## RESULTS

The sample of the pre-test corresponded to 17 older adults, 65% male, at an average age of 70.5, and with up to four years of study.

The general and referential equivalence of meanings between the original version and the reverse translations (RT1 and RT2) varied from 50% to 100% (Table 1).

**Table 1:** Comparison of general and referential meanings between the two reverse translations and the original version of the Vulnerability to Abuse Screening Scale *(*VASS).

Item	Original	T1 – RT1	R (%)	G	T2 – RT2	R (%)	G
1	Are you afraid of anyone in your family?	Are you afraid of anyone in your family?	100	UN	Are you afraid of someone in your family?	50	DC
2	Has anyone close to you tried to hurt you or harm you recently?	Has anyone close to you tried to hurt you or offend you recently?	90	SC	Has anyone close to you tried to hurt or offend you recently?	90	SC
3	Has anyone close to you called you names or put you down or made you feel bad recently?	Has anyone close to you called you names, humiliated you or made you feel bad recently?	95	UN	Has anyone close to you cursed, humiliated you or made you feel bad recently?	85	SC
4	Do you have enough privacy at home?	Do you have enough privacy in your home?	90	SC	Do you have enough privacy at your home?	100	UN
5	Do you trust most of the people in your family?	Do you trust most of the people in your family?	100	UN	Do you trust most people in your family?	100	UN
6	Can you take your own medication and get around by yourself?	Can you take your medication and move around by yourself?	100	UN	Are you able to take your medication and to get around by yourself?	100	UN
7	Are you sad or lonely often?	Do you frequently feel sad or lonely?	100	UN	Do you often feel sad or lonely?	100	UN
8	Do you feel that nobody wants you around?	Do you feel like no one wants you near them?	95	UN	Do you feel nobody wants you around?	100	UN
9	Do you feel uncomfortable with anyone in your family?	Do you feel embarrassed by anyone in your family?	90	SC	Do you feel embarrassed with anyone in your family?	90	SC
10	Does someone in your family make you stay in bed or tell you you’re sick when you know you’re not?	Does anyone in your family make you stay in bed or say that you are sick when you know you’re not?	100	UN	Does anyone in your family oblige you to stay in bed or say that you are ill, when you know you are not?	100	UN
11	Has anyone forced you to do things you didn’t want to do?	Has anyone ever forced you to do things you didn’t want to do?	100	UN	Has anyone forced you to do things you did not want to?	100	UN
12	Has anyone taken things that belong to you without your OK?	Has anyone ever taken things that belong to you without your consent?	100	UN	Has anyone got your belongings without your permission?	100	UN

T1: first translation to Portuguese; RT1: first reverse English-Portuguese translation; T2: second translation to Portuguese; RT2: second reverse translation; R: referential meaning (% of similarity to the original version); G: general meaning; UN: unchanged; SC: slightly changed; DC: drastically changed

The general meaning of the reverse translations (RT1 and RT2) was satisfactory, with most questions having unchanged meanings (eight questions – 66.5%) in both versions. Item “1” in RT2 was problematic, as inappropriate use of the “someone” pronoun drastically changed its meaning. The second and ninth items were slightly changed in both versions. The independent reverse translations of item “2” presented the idea that the term “harm” in English could mean “offend”. Despite being synonyms, we noticed in this adaptation that the word “harm” would be nearer to the sense of aggravation or damage, as proposed in the original translation of the instrument. Thus, the word “harm” was used literally in the pre-test version.

For item “9”, we preferred RT2 because of the idea of a feeling of discomfort caused “by anyone in your family” and not “by someone”. Thus, we maintained the same criteria used to judge item “2”, and the second reverse translation proved more coherent with the meaning of the question.

Both reverse translations were balanced in terms of meaning percentages, with RT2 slightly more adequate than RT1 in the referential sense. Consequently, we chose more items from RT2 (eight items) when creating the synthesized version (Table 1).

During the pilot study, the questionnaire was tested and well accepted by the interviewees. It was easily applied, with an average of two minutes for completion of the questionnaire.

Differences in terms of the clarity and objectivity of the instrument were discussed among the older people. We analyzed the suggested changes and observed the pertinence of the adjustments for the purpose of clarity and objectivity.

Items 4, 7, 8, 9, and 11 were changed to use words that were easier to understand, and to include “Mr.” and “Mrs./Ms.” Instead of “you” in order to address the interviewee respectfully. We also included articles to avoid long phrases or repeated ideas. The final translation of the scale is shown in Table 2.

**Table 2:** Origin of the translated items, synthesized version used in the pre-test, and final version of Vulnerability to Abuse Screening Scale (VASS).

Path from the first synthesized version (origin of the items) until the final version after semantic equivalence			

Item	Translation original	Version submitted to semantic equivalence	Final version
1	*Você tem medo de alguém da sua família? (T2)*	*O(A) senhor(a) tem medo de alguém da sua família?*	*O(A) Sr.(a) tem medo de alguém da sua família?*
2	*Alguém próximo a você tentou te machucar ou te ofender recentemente? (T2)*	*Alguma pessoa próxima ao(a) senhor(a) tentou te machucar ou te ofender recentemente?*	*Alguma pessoa próxima ao(a) Sr.(a) tentou machuca-lo(a) ou prejudica-lo(a) recentemente?*
3	*Alguma pessoa próxima a você te xingou, humilhou ou fez com que se sentisse mal recentemente? (T1)*	*Alguma pessoa próxima ao(a) senhor(a) te xingou, humilhou ou te fez se sentir mal recentemente?*	*Alguma pessoa próxima ao(a) Sr.(a) te xingou, humilhou ou fez o(a) Sr.(a) se sentir mal recentemente?*
4	*Você tem privacidade suficiente em sua casa? (T1 e T2)*	*O(A) senhor (a) tem privacidade suficiente em sua casa?*	*Na sua casa, o seu espaço e privacidade são respeitados?*
5	*A você confia na maior parte das pessoas de sua família? (T1)*	*O(A) senhor(a) confia na maioria das pessoas de sua família?*	*O(A) Sr.(a) confia na maioria das pessoas da sua família?*
6	*Você pode tomar seus medicamentos e se locomover sem ajuda? (T1)*	*O(A) senhor(a) consegue tomar sua medicação e locomover-se sozinha?*	*O(A) Sr.(a) consegue tomar sua medicação e andar para lugares que precisa ir sem a ajuda de alguém?*
7	*Você se sente triste ou solitária com frequência? (T1)*	*O(A) senhor(a) se sente triste ou solitária (o) com frequência?*	*O(A) Sr.(a) se sente, na maioria das vezes, triste ou solitário(a)?*
8	*Você sente que ninguém te quer por perto? (T2)*	*O(A) senhor(a) sente que ninguém te quer por perto?*	*O(A) Sr.(a) se sente rejeitado(a) por pessoas que são próximas ou íntimas do(a) Sr.(a)?*
9	*Você se sente constrangida frente a alguma pessoa de sua família? (T2)*	*O(A) senhor(a) se sente desconfortável quando está perto de alguma pessoa da sua família?*	*O(A) Sr.(a) se sente incomodado(a) quando está perto de alguém da sua família?*
10	*Alguém da sua família te faz ficar na cama ou diz que você está doente quando você sabe que você não está? (T2)*	*Alguém da sua família te obriga a ficar na cama ou te diz que está doente quando o(a) senhor(a) sabe que não está?*	*Alguém da sua família te obriga a ficar na cama ou fala que o(a) Sr.(a) está doente quando o(a) Sr.(a) sabe que não está?*
11	*Alguém já te forçou a fazer coisas que você não queria fazer? (T2)*	*Alguém já te forçou a fazer alguma coisa que o(a) senhor(a) não queria fazer?*	*Alguém já o(a) obrigou a fazer coisas que o(a) Sr.(a) não queria fazer?*
12	*Alguém já pegou coisas que te pertencem sem o seu consentimento? (T2)*	*Alguém já pegou coisas que te pertencem sem o seu consentimento?*	*Alguém já pegou coisas que te pertencem sem a sua permissão?*

T1: first translation to Portuguese; T2: second translation to Portuguese

The sample for the psychometric tests corresponded to 151 elderly people, with an average age of 72.05 years (DP = 6.96), minimum of 60 years, and maximum of 92 years. The average age of the women was slightly lower than that of the men (71.6 *versus* 73.7 years). The sample was predominantly female (76.2%) and presented MEEM scores above 12 points with an average of 25.3 (DP = 2.68). The subjects were active and had a 20.0 (DP = 0.13) score average in instrumental activities of daily life (Lawton and Brody^22^) and 3.75 (DP = 0.26) on the Geriatric Depression Scale (GDS)^1^, for which the maximum value was 13.

When we compared the VASS scores, categorized by presence (score ≥ 3) or absence (score ≤ 3) of vulnerability to abuse, there were statistically significant differences in the self-perception of health (p = 0.002), depressive symptoms (p = 0.001), and presence of rheumatism (p = 0.003). There were no statistically significant differences between the sexes. Table 3 presents the univariate analysis of the sample.

**Table 3:** Univariate analysis of the sample with regard to the presence or absence of vulnerability to abuse, 2014. (N = 151)

Sample characteristics		VASS < 3		VASS ≥3		p*
	
		n	%	n	%	
Sociodemographic						

Sex	Male	22	23.9	14	23.7	0.979
	Female	70	76.1	45	76.3	
Education (years)	≤ 4	42	45.7	23	39.0	0.632
	5-8	35	38.0	27	45.8	
	≥ 9	15	16.3	9	15.3	
Income	≤ 3 salaries	80	87.0	53	91.4	0.405
	> 3 salaries	12	13.0	5	8.6	
Marital status	With spouse	25	27.2	17	28.8	0.826
	Without spouse	67	72.8	42	71.2	
Lives alone?	Yes	20	21.7	19	32.2	0.152
	No	72	78.3	40	67.8	

Clinical conditions						

Health perception	Good	65	71.4	27	45.8	0.002
	Poor	26	28.6	32	54.2	
HAS	No	24	26.1	17	28.8	0.713
	Yes	68	73.9	42	71.2	
DM	No	71	77.2	45	76.3	0.898
	Yes	21	22.8	14	23.7	
Cardiac disease	No	82	89.1	45	76.3	0.035
	Yes	10	10.9	14	23.7	
Rheumatism	No	66	71.7	28	47.5	0.003
	Yes	26	28.3	31	52.5	
Depression	No	81	88.0	43	72.9	0.028
	Yes	11	12.0	16	27.1	
Internment	Yes	14	15.2	9	15.3	0.995
	No	78	84.8	50	84.7	
Polypharmacy (medication)	≥ 5	23	25.0	17	28.8	0.604
	< 5	69	75.0	42	71.2	

Vulnerability factors						

Bedridden	Yes	13	14.1	12	20.3	0.317
	No	79	85.9	47	79.7	
MEEM	Normal	41	44.6	30	50.8	0.450
	Cognitive decline	51	55.4	29	49.2	
GDS	< 5	75	81.5	28	47.5	0.001
	≥ 5	17	18.5	31	52.5	
AVDI	Independent	58	63.0	30	50.8	0.138
	Dependent	34	37.0	29	49.2	

VASS: Vulnerability to Abuse Screening Scale; HAS: systemic arterial hypertension; DM: diabetes mellitus; MEEM: Mini Mental State Exam; GDS: Geriatric Depression Scale; AVDI: instrumental daily activities

* Comparisons through the Fisher test with significance level of 0.05.

The Kaiser-Meyer-Olkin test indicated a median yet acceptable adaptation of the sample, with a value of 0.64 and satisfactory adaptation for the factorial analysis by the Bartlett sphericity test, which showed an identity matrix with statistical significance (p < 0.002).

Three factors met the Kaiser criteria, for which dimensions were extracted with self-value higher than or equal to one. However, based on the assumption that factors with higher explanation potential for the subjacent dimension may contribute to the explanation of the model, we opted to keep the fourth dimension. This was because it strengthened the idea of the general construct of the instrument, agreeing with the model proposed by the authors of the VASS^23^, and had a self-value very close to one (SV = 0.97).

We performed a factorial analysis via principal components with all 12 of the items in the scale. Initially, we found a low factor load of variable 12, in addition to the exclusive allocation of only one item in a factor (variable five in factor four). To adjust the model, we performed a new analysis without this variable.

In the second model, we found changes in the allocation of the factors and the items, which did not compromise the main idea of the constructs, remaining the same designation for the four factors, namely: vulnerability, discouragement/depression, coercion, and dependence. The factor loads varied from 0.57 to 0.92, with communalities between 0.62 and 0.93.

As in the original study, factor “1” preserved the “vulnerability” construct, and came in first place, carrying items 8 and 9, in addition to variables 1, 2, and 3. The communalities of this factor varied between 0.62 and 0.88 and corresponded to 28% of the explained variance.

The “discouragement/depression” construct moved positions in this study. This was the third factor in the original study, represented by variables 7, 8, and 9, and came second in this analysis, aggregating questions 4 and 5. The third dimension corresponded to the “coercion” construct (completed by variables 10 and 11), and preserved the idea of stress due to potential violence experienced, as in the original study. The “dependence” dimension was the second factor in the original study and explained 32% of the variance in the validation of the VASS in 2003. In this analysis, it came in last place, with a higher explanatory potential (35% of the explained variance), despite one variable less (items 5 and 6).

All factors in all items were determined (Table 4).

**Table 4:** Factorial analysis of the Portuguese version of the Vulnerability to Abuse Screening Scale (VASS).

Items	Factor 1	Factor 2	Factor 3	Factor 4	Communalities (%)
			
	Vulnerability	Discouragement/Depression	Coercion	Dependence	
VASS3	0.92	0.17	0.04	-0.02	0.88
VASS2	0.86	0.28	-0.07	0.02	0.82
VASS9	0.66	-0.13	0.12	0.40	0.63
VASS8	0.64	0.17	0.29	0.31	0.62
VASS1	0.57	0.06	0.28	0.57	0.74
VASS7	0.05	0.79	0.25	0.16	0.71
VASS4*	-0.27	0.73	-0.19	0.01	0.65
VASS10	-0.12	0.46	0.82	0.18	0.93
VASS11	0.38	0.15	0.82	-0.07	0.84
VASS6*	-0.06	-0.09	-0.04	0.88	0.79
VASS5*	-0.26	-0.50	0.38	0.59	0.80
Self-values	4.319	1.772	1.346	0.979	
% explained variance	28%	14%	16%	16%	

* Items with score points from negative answers.

The internal consistency of the global VASS scale obtained a KR-20 of 0.69 (p < 0.001), showing good consistency, especially considering the brief structure of the instrument.

For the four constructs, reliability was considered good to moderate, with KR-20 values of 0.715, 0.32, 0.51, and 0.35.

In the “vulnerability” subscale, the simultaneous removal of each item reduces its internal consistency. We identified a similar finding when analyzing the impact of the removal of items from this dimension on the scale total – in other words, the removal of all items from this subscale reduces the internal consistency of the VASS global construct.

As the remaining factors were composed of only two variables, the item-total correlations were emitted through the removal of each item with the evaluation of the impact on the KR-20 value of the VASS global score. In this regard, the internal consistency in each case either decreased or remained the same, showing that the items are correlated and help explain the VASS global construct idea.

The intra-observer reliability considered three instances: the stability of the answer to each item; for the global scale given the sum of the points attributed on a scale from zero to 12; and for each subscale or subjacent dimension of the VASS. For each item analyzed individually, the K values varied between 0.37 and 0.93 (p < 0.001), reflecting good and adequate concordance. Only questions six and 10 obtained lower concordance estimates. The result of the Kp statistic for the total of the four constructs of the VASS was 0.97 (p < 0.001), proving that the global instrument has substantial reliability. For each factor or subscale, the results found varied from excellent (0.955, 0.890) to moderately reliable (0.736 and 0.561) (Table 5).

**Table 5:** Internal consistency and intra-observer reliability (reproducibility) of the VASS adapted to Brazilian culture.

Constructs	Items	Internal consistency		Reproducibility			K^c^	p
	
		KR-20	(KR-20_KR-1_)^a^	K^b^		p		
Vulnerability		0.71	Dimension - VASS				0.955	< 0.001
	Vass3		0.62-0.64	0.825		0.000		
	Vass2		0.66-0.64	0.866		0.000		
	Vass9		0.69-0.66	0.731		0.000		
	Vass8		0.65-0.64	0.936		0.000		
	Vass1		0.68-0.65	0.729		0.000		
Discouragement/Depression^d^		0.32	VASS				0.890	< 0.001
	Vass7		0.69	0.923		0.000		
	Vass4		0.67	0.695		0.000		
Coercion^e^		0.51	VASS				0.736	< 0.001
	Vass10		0.69	0.370		0.000		
	Vass11		0.68	1.000		0.000		
Dependence^e^		0.35	VASS				0.561	< 0.001
	Vass5		0.67	1.000		0.000		
	Vass6		0.68	0.492		0.000		
	General	0.6904					0.927	< 0.001

^a^ Change in internal consistency after removal of item.

^b^ Simple Kappa for each item.

^c^ Kappa with quadratic weighting for each construct.

^d^ Change in internal consistency after removal of item, considering the score of the dimension and the global VASS score.

^e^ Change in internal consistency considering the global score of the scale.

## DISCUSSION

This transcultural adaptation verified the semantic equivalence of the VASS for Brazilian culture. Observance of recommendations for the transcultural translation and adaptation of the scale^2^ ^,^ ^7^ ^,^ ^13^, with rigorous appliance of the methodology, was fundamental in problem solving, proving to be a more appropriate method than simple translation of the instrument.

By comparing the findings of transcultural adaptation of the VASS in this study with those found by Maia and Maia^11^, we encountered similarities regarding general and referential meaning. For general meaning, this study was more successful in RT2, with nine items against eight unchanged items presented by Maia and Maia. As for the connotative aspect, only one item was problematic in this study (item 1), which occurred with item 3 in the study by Maia and Maia. The semantic equivalence process in this investigation resulted in an unambiguous questionnaire that is linguistically adapted to Brazilian culture.

As for the construct validity of the VASS, the dimensional analysis with four factors proves the findings of Schofield and Mishra^23^, and Schofield et al.^24^ We adjusted the factorial loads, since all the items had loads in exclusive dimensions varying from 0.57 to 0.92, with satisfactory communalities (0.62 to 0.93). The results were better than those found in the original study^24^ and in the later validation analysis of the VASS, which showed maximum communalities of 0.60 and 0.77, respectively, as well as a maximum factorial load of 0.76 and 0.87 for the “vulnerability” dimension, which had greater factorial loads in both analyses^23^ ^,^ ^24^.

In this regard, the performance of this study seems to have surpassed the findings of Reichenheim et al. in the adaptation process of the HS- EAST^19^ for Brazilian culture.

The factors were also repositioned in the second phase of the VASS application in the cohort studied in Australia, after three years of follow-up from the first interview. On that occasion, despite the factors having been placed in distinct positions, the original items remained in them^24^.

In addition to this repositioning, the composition of the factors was also different. The “vulnerability” factor aggregated the highest number of variables (five of the 11 items), and its composition maintained the original values, but aggregated two others (items 8 and 9), which belonged to the discouragement/depression factor. Despite the changes, this allocation seems to have been pertinent, since variables 8 (“Do you feel that nobody wants you around?”) and 9 (“Do you feel uncomfortable with anyone in your family?”) presented, in the fifth phase of semantic equivalence, the impression that they are more closely associated with situations of interpersonal conflict than with depression. This reinforces the findings of the VASS validation in Australia, which showed high face validity for the “vulnerability” factor as a measure of susceptibility to mistreatment, as well as a strong association with stressful or abusive events, especially in relations with a partner/spouse, children, and other family members^23^.

We highlight that these items are formulated exactly as the HS EAST instrument, and, thus, both the validation conducted by Neale et al.^15^ and the research regarding adaptation to the Brazilian context by Reichenheim et al.^19^ supported similar ideas in the construct that represents them (“abuse and violation of personal rights”).

Another possible explanation for the repositioning of the items is the size and characterization of the sample of this study, represented by active older adults who participate in health promotion activities, and who have better health indicators than the samples of the original VASS studies.

The greatest change in the structure of the factors occurred in the second dimension, “discouragement/depression”, which, in the original version, was composed of variables 7 (“Are you sad or lonely often?”), 8 (“Do you feel that nobody wants you around?”), and 9 (“Do you feel uncomfortable with anyone in your family?”). The current version kept item 7 and added question 4 (“Do you have enough privacy at home?”).

The internal consistency of the general VASS score (KR-20 = 0.69) is considered adequate and good. There are no reliability analyses, considering the sum of the items in the scale in the studies of the main author^21^ ^,^ ^24^.

When comparing reliability between the global VASS index and the similar instrument it was based on (HS EAST), both in its context of origin and in the Brazilian one^19^, the VASS values were higher (α = 0.29 and KR-20 = 0.64).

The isolated removal of each of the 12 items decreased or maintained the internal consistency of the global scale, indicating that the variables contribute to the explanation of the general construct. It was not possible to compare these data with the previous VASS studies, as this analysis was presented only in this study.

The vulnerability construct is the most substantial (KR-20 = 0.71), gaining explanation power and better reliability in relation to the values found in studies by Schofield et al.^23^ and Schofield and Misrha^24^, who observed lower values of Cronbach alpha (α), which were 0.55 and 0.45, respectively.

Despite the lower number of items, in the remaining subscales (discouragement/depression, coercion, and dependence), the internal consistency values remained very close to those found in previous studies, with the exception of the “dependence” factor, which had a lower score in this study (KR-20 = 0.32), contrasting with previous studies (α = 0.52 and 0.74) ^23^ ^,^ ^24^.

As for the reproducibility of the VASS, we can safely affirm that the general score is satisfactorily reliable (Kp = 0.92) and has better performance than the HS EAST^20^ (Kp = 0.70).

Until now in Brazil, no methods qualify as a gold standard for approaching domestic violence towards elderly people. The limitations of this study include our inability to test the sensibility and specificity of the triage instrument, and the fact that we did not perform a confirmatory factorial analysis using, for example, the structural equations method, since this analysis method requires a larger sample than that used in this study.

However, the VASS behaved acceptably in terms of dimensionality, and was coherent with the original proposal. Thus, there do not seem to be any contraindications for its use, except in environments of primary health care or reference centers for the elderly.

## Footnotes

a Minayo MCS. Violência contra idosos: o avesso do respeito à experiência e à sabedoria*.* Brasília (DF): Secretaria Especial de Direitos Humanos; 2005.
